# Analysis and projections of disease burden for different risk factors and sexes of ischemic stroke in young adults in China

**DOI:** 10.1038/s41598-024-63920-0

**Published:** 2024-06-10

**Authors:** Ying Gao, Kangding Liu, Shaokuan Fang

**Affiliations:** Department of Neurology, Neuroscience Centre, The First Hospital of Jilin University, No. 1 Xinmin Street, Chaoyang District, Changchun, 130021 Jilin Province People’s Republic of China

**Keywords:** Ischemic stroke, Young adults, Risk factors, Metabolic risk factors, The Global Burden of Disease, Neurology, Neurological disorders, Weight management

## Abstract

To estimate the rate of death, and disability-adjusted life years (DALYs) and project the disease burden of ischemic stroke due to relevant risk factors in young adults age 20–49 years by sex in China. Data from the Global Burden of Diseases, Injuries, and Risk Factors Study (GBD) 2019 were used. The age-standardized mortality (ASMR), age-standardized DALYs rate (ASDR), and estimated annual percentage changes (EAPC) were calculated to evaluate the temporal trends from 1990 to 2019. We also used the NORDPRED model to predict ASMR for ischemic stroke due to related risk factors in Chinese young adults over the next 10 years. From 1990 to 2019, the general age-standardized mortality [from 2.39 (1.97 to 2.99) in 1990 to 1.8 (1.41 to 2.18) in 2019, EAPC = − 1.23] and DALYs rates (from 171.7 (140.34 to 212.36) in 1990 to 144.4 (114.29 to 177.37) in 2019, EAPC = − 0.86) decreased for ischemic stroke in young adults in China. ASMR and ASDR decreased for all level 1 risk factors (including behavioral, environmental/occupational, and metabolic) from 1990 to 2019, with the slightest decrease for metabolic risks [ASMR from 1.86 (1.39 to 2.41) in 1990 to 1.53 (1.15 to 1.92) in 2019, ASDR from 133.68 (99.96 to 173.89) in 1990 to 123.54 (92.96 to 156.98) in 2019] and the largest decrease for environmental/occupational risks [ASMR from 1.57 (1.26 to 1.98) in 1990 to 1.03 (0.78 to 1.29) in 2019, ASDR from 110.91 (88.44 to 138.34) in 1990 to 80.03 (61.87 to 100.33) in 2019]. In general, high body-mass index, high red meat intake, and ambient particulate matter pollution contributed to the large increase in ASMR and ASDR between 1990 and 2019. Significant reductions in ASMR and ASDR were observed in low vegetables intake, household air pollution from solid fuels, lead exposure, and low fiber intake. In addition, there were sex differences in the ranking of ASMR attributable to risks in ischemic stroke. The disease burden of ischemic stroke attributable to relevant risk factors in young adults in China is greater and has a faster growth trend or a slower decline trend in males than in females (except for secondhand smoke). The apparent increasing trend of ASMR attributable to high fasting plasma glucose, high systolic blood pressure, high body-mass index, and high red meat intake was observed in males but not in females. The projected analysis showed an increasing trend in ASMR between 1990 and 2030 for all specific metabolic risks for males, but a decreasing trend for females. ASMR attributable to ambient particulate matter pollution showed an increasing trend from 1990 to 2030 for both males and females. The burden of ischemic stroke in young adults in China showed a downward trend from 1990 to 2019. Specific risk factors associated with the burden of ischemic stroke varied between the sexes. Corresponding measures need to be developed in China to reduce the disease burden of ischemic stroke among young adults.

## Introduction

Stroke is one of the leading causes of death and long-term disability in low- and middle-income countries^1^. Ischemic stroke accounts for 70 percent of all strokes, and the total number of ischemic stroke-related deaths in 2019 reached 3.29 million, accounting for more than half of all stroke deaths^2^. In China, there are more than 2 million new cases of stroke each year, and the burden is expected to increase further due to demographic changes and increased risk factors^3^. Although stroke is thought to be common in older adults, recent studies have shown an increase in the incidence of ischemic stroke in low- and middle-income countries^4^. Economic development, social ideology, and demographic changes have led to different changes in metabolic and behavioral risk factors for ischemic stroke, such as smoking, high body-mass index, high fasting blood glucose, and high systolic blood pressure^2,5^. Studies of changes in the risk factors for ischemic stroke can help guide disease prevention and reduce the economic and disease burdens.

The most widely accepted definition of “young adult stroke” refers to any type of stroke in the age between 18 to 49 years^6^. Stroke in younger people has a smaller disease burden than stroke in older people, but the economic impact can be greater due to the loss of productivity caused by physical disability after stroke^6–8^. Understanding trends in the risk factors for ischemic stroke in young adults can help to specify appropriate prevention guidelines, improve the level of primary prevention of ischemic stroke, and reduce the burden on families and society. However, no research has estimated the mortality, and disability-adjusted life years (DALYs) of ischemic stroke in young adults attributable to different risk factors in China.

In this study, we used ischemic stroke data of China from 1990 to 2019 from the Global Burden of Diseases, Injuries, and Risk Factors Study (GBD) 2019 to examine temporal trends and sex differences in age-standardized mortality rates (ASMR) and DALYs rates (ASDR) for stroke among young adults due to different risk factors. In this study, we aimed to: (1) describe changes in the disease burden of ischemic stroke in young adults in China due to different risk factors from 1990 to 2019; (2) show the disease burden of ischemic stroke in young adults in China due to different risk factors by sex differences; (3) project ASMR for ischemic stroke due to several significant risk factors in Chinese adults.

## Methods

### Data sources

We obtained data about deaths, DALYs and age specific cases relating to ischemic stroke and attributable risk factors in China during 1990 to 2019 from GBD 2019 (https://ghdx.healthdata.org/gbd-results-tool), which provided a systematic approach to collecting data such as disease deaths and DALYs. Data on stroke mortality in GBD 2019 for China is mainly extracted from the Chinese surveillance system, which has provided provincial representative mortality data for decades^9^. Details of the description and methods used to evaluate the risk factor data have been described in previous publications^10^. In this study, we estimated the three risk factors at level 1 and 20 specific risk factors. The predicted Chinese population was obtained from the GBD database (https://ghdx.healthdata.org/record/ihme-data/global-population-forecasts-2017-2100).

### Definitions

Ischemic stroke is identified as all vascular events that lead to restricted blood flow to brain tissue resulting in infarction, including atherosclerotic and thromboembolic factors but excluding factors that cause intracranial hemorrhage^11^. Based on the availability of data and the incidence and mortality of ischemic stroke under the age of 20 is low, the definition of young adults in our study is ages between 20 to 49 years.

### Measurements

GBD 2019 estimated attributable mortality, years of life lost (YLLs), years of life lived with disability (YLDs), and DALY s for 87 risk factors and combinations of risk factors. YLLs due to premature mortality were calculated by multiplying the number of deaths in each age group by the remaining life expectancy in that age group using the GBD standard life table. YLDs were years lived with short-term or long-term health loss weighted by the severity of the disability. DALYs were calculated as the sum of YLLs and YLDs, also defined as years of healthy life lost^10^. The Bayesian meta-regression method (DisMod-MR) are used to analyzed the disease burden of ischemic stroke attributable to risk factors, including the following several steps: (1) identifying the inclusion of risk-outcome pairs; (2) estimation of relative risk as functional exposure; (3) estimation of exposures for each risk by age, sex, location, and year; (4) identification of the theoretical minimum risk exposure level (TMREL) and the counterfactual exposure; (5) estimation of attributable burden and population attributable fractions (PAFs); and (6) estimation of the deaths and DALYs attributable to various combinations of risk factors^10^.

### Standard Protocol Approvals, Registrations, and Patient Consents

This study was approved by the medical ethics committee of First Hospital of Jilin University, China. The study followed the Guidelines for Accurate and Transparent Health Estimation Reporting for Population Health Research, and informed consent was not required.

### Statistical Analysis

The age-standardized rates (ASR) were calculated using the GBD world population age standard as a reference. Direct standardization yields an age-adjusted rates, which are weighted averages of age-specific rates. The weights are intended to represent the relative age distribution. The ASR is calculated by the following equation^6,12^:

$${\text{ASR}} = \frac{{\mathop \sum \nolimits_{i = 1}^{A} a_{i} \omega_{i} }}{{\Sigma_{i = 1}^{A} \omega_{i} }} \times 100,000$$

where ${a}_{i}$ and ${\omega }_{i}$ denote age-specific rates and the number of persons (or weight) in the same age subgroup of the chosen reference standard population (where $i$ denotes the $i$th age class), respectively.

The estimated annual percentage changes (EAPC) and its 95% confidence interval (CI) are calculated using a log-linear regression as follows: Ln (ASR) = α + βx + ε, x is the year, and β is the regression coefficient. EAPC was calculated as 100 × [exp(β) − 1], and the 95% CI of the EAPC was generated based on the standard error generated by the log-linear regression. The ASR has been considered increased if the EAPC and its 95% CI were > 0, decreased if they were < 0, if the 95% CI included 0, the rate was considered to be a relatively stable trend^13^. In order to more clearly show the changes in the ranking of risk factors, we produced risk factor ranking charts for 1990, 2005, and 2019. To predict the number of deaths and ASMR from 2020 to 2030, a log-linear age-period-cohort model, which levels off the exponential growth and limits the linear trend projection, is fitted to recent trends. This model is implemented in R through the package NORDPRED and performs well in predicting disease trends, as demonstrated in previous studies^14–16^ The rates are predicted by extrapolating over three to five-year periods, and the quantities are determined by model goodness-of-fit. We extrapolate the most recent 10-year linear trend, attenuating the slope by 25% and 50% in the second and third prediction periods, and by 75% in the fourth and fifth periods, as recommended by the authors following empirical validation^17^. All analyses were performed using R version 4.1.3.

### Consent statement

The requirement for informed consent was waived by the Ethics Committee of the First Hospital of Jilin University due to the GBD is a publicly available database and all participants’ data were anonymous.

## Results

### ASMR and ASDR for ischemic stroke in young adults in China showed a decreasing trend

**Figure 1:**
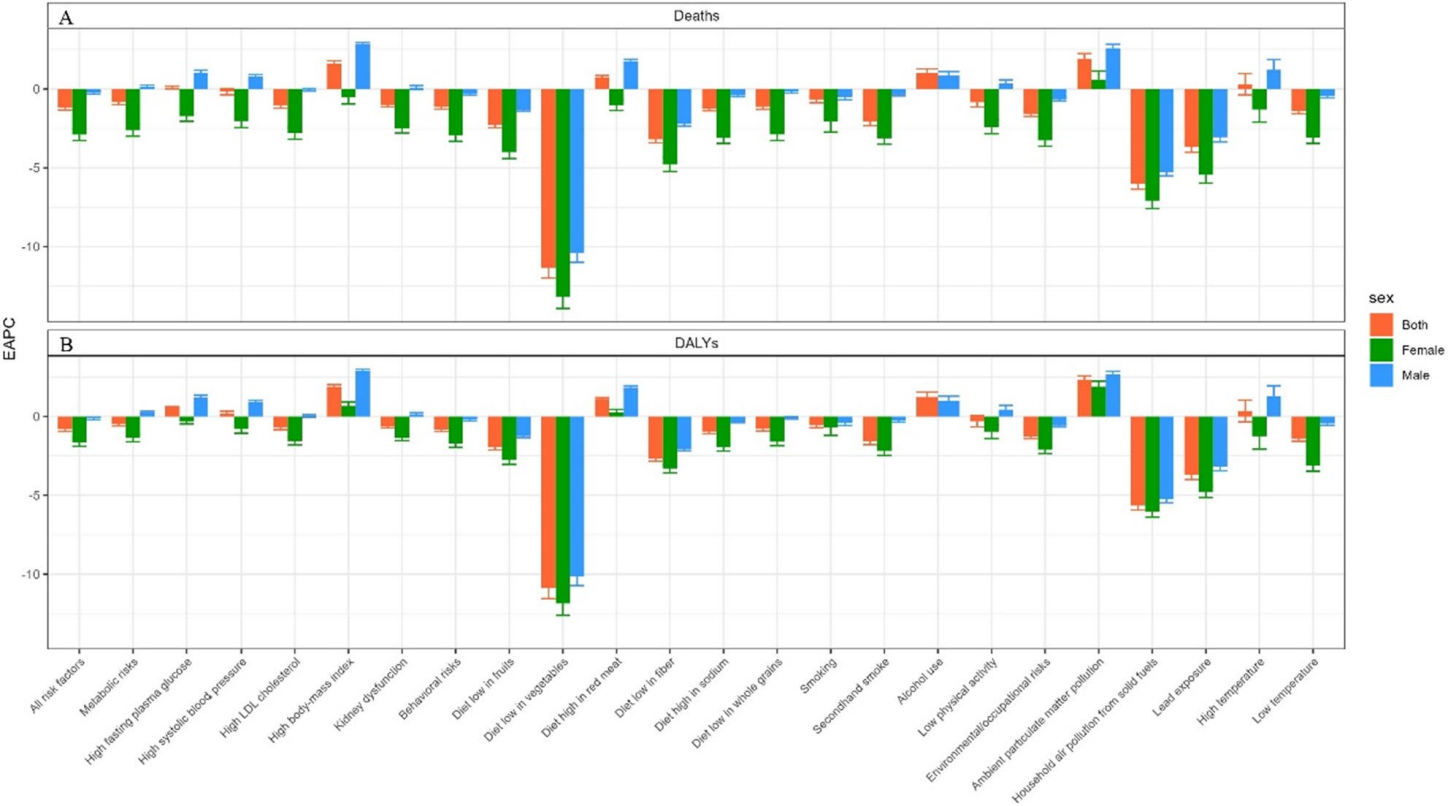
Estimated annual percent change (EAPC) of general and specific risk factors age-standardized deaths (**A**), and DALYs (**B**) for ischemic stroke in young adults aged 20–49 years by sex in China.

Overall, the ASMR and ASDR of ischemic stroke in young adults in China gradually decreased from 1990 to 2019. ASMR for ischemic stroke in young adults was 2.52 per 100 000 population in 1990 and 1.87 per 100,000 population in 2019, and EAPC showed a decreasing trend from 1990 to 2019 [EAPC = − 1.23 (− 1.39 to − 1.07)] (Table S1). The EAPC of ASMR for metabolic risks, behavioral risks, and Environmental/occupational risks was − 0.83 (− 1 to − 0.67), − 1.14 (− 1.28 to − 0.99), and − 1.58 (− 1.75 to − 1.42), respectively (Table 1 and Fig. 1A).

**Table 1:** Trends in ASMR for ischemic stroke in young adults in China from 1990 to 2019 for all risk factors.

	1990		2019		EAPC		
	ASMR for male per 105 (95% CI)	ASMR for female per 105 (95% CI)	ASMR for male per 105 (95% CI)	ASMR for female per 105 (95% CI)	Both No. (95% CI)	Male No. (95% CI)	Female No. (95% CI)
All risk factors	2.77 (2.12 to 3.76)	1.97 (1.54 to 2.53)	2.55 (1.84 to 3.25)	1.01 (0.76 to 1.31)	− 1.18 (− 1.33 to − 1.02)	− 0.22 (− 0.31 to − 0.13)	− 2.89 (− 3.27 to − 2.51)
Metabolic risks	2.13 (1.48 to 2.99)	1.55 (1.11 to 2.13)	2.18 (1.5 to 2.86)	0.87 (0.6 to 1.17)	− 0.83 (− 1 to − 0.67)	0.16 (0.06 to 0.25)	− 2.61 (− 2.99 to − 2.22)
High fasting plasma glucose	0.28 (0.08 to 0.64)	0.18 (0.05 to 0.4)	0.34 (0.09 to 0.74)	0.12 (0.03 to 0.27)	0.09 (0.01 to 0.18)	1.03 (0.87 to 1.19)	− 1.73 (− 2.05 to − 1.4)
High systolic blood pressure	1.2 (0.49 to 2.16)	0.8 (0.23 to 1.55)	1.46 (0.7 to 2.25)	0.53 (0.18 to 0.92)	− 0.17 (− 0.37 to 0.03)	0.78 (0.64 to 0.92)	− 2.03 (− 2.45 to − 1.6)
High LDL cholesterol	1.45 (0.9 to 2.07)	1.09 (0.71 to 1.55)	1.4 (0.87 to 2)	0.58 (0.37 to 0.81)	− 1.05 (− 1.21 to − 0.89)	− 0.04 (− 0.13 to 0.05)	− 2.81 (− 3.18 to − 2.43)
High body-mass index	0.4 (0.09 to 0.94)	0.32 (0.08 to 0.72)	0.86 (0.37 to 1.49)	0.32 (0.13 to 0.59)	1.62 (1.45 to 1.79)	2.84 (2.73 to 2.95)	− 0.52 (− 0.95 to − 0.1)
Kidney dysfunction	0.24 (0.16 to 0.35	0.23 (0.16 to 0.32)	0.23 (0.15 to 0.32)	0.12 (0.08 to 0.17)	− 1.01 (− 1.13 to − 0.89)	0.09 (− 0.05 to 0.22)	− 2.51 (− 2.79 to − 2.23)
Behavioral risks	2.32 (1.74 to 3.13)	1.37 (1.01 to 1.82)	2.11 (1.51 to 2.71)	0.69 (0.49 to 0.93)	− 1.14 (− 1.28 to − 0.99)	− 0.29 (− 0.38 to − 0.2)	− 2.95 (− 3.31 to − 2.59)
Diet low in fruits	0.4 (0.1 to 0.76)	0.29 (0.07 to 0.56)	0.28 (0.06 to 0.57)	0.11 (0.02 to 0.22)	− 2.28 (− 2.47 to − 2.09)	− 1.3 (− 1.4 to − 1.2)	− 4 (− 4.41 to − 3.58)
Diet low in vegetables	0.14 (0.01 to 0.27)	0.1 (0.01 to 0.19)	0.01 (0 to 0.02)	0 (0 to 0.01)	− 11.34 (− 12 to − 10.68)	− 10.39 (− 11 to − 9.78)	− 13.17 (− 13.93 to − 12.41)
Diet high in red meat	0.43 (0.12 to 0.75)	0.32 (0.09 to 0.55)	0.66 (0.33 to 0.96)	0.26 (0.13 to 0.39)	0.75 (0.64 to 0.87)	1.77 (1.64 to 1.89)	− 1.03 (− 1.35 to − 0.71)
Diet low in fiber	0.34 (0.07 to 0.67)	0.26 (0.06 to 0.48	0.17 (0.02 to 0.43)	0.08 (0.01 to 0.18)	− 3.19 (− 3.41 to − 2.97)	− 2.21 (− 2.35 to − 2.08)	− 4.79 (− 5.23 to − 4.36)
Diet high in sodium	0.85 (0.35 to 1.49)	0.49 (0.18 to 0.9)	0.74 (0.28 to 1.31)	0.24 (0.08 to 0.44)	− 1.25 (− 1.38 to − 1.12)	− 0.4 (− 0.49 to − 0.31)	− 3.1 (− 3.45 to − 2.75)
Diet low in whole grains	0.33 (0.07 to 0.55	0.24 (0.05 to 0.38)	0.31 (0.07 to 0.52)	0.12 (0.03 to 0.2)	− 1.13 (− 1.3 to − 0.97)	− 0.16 (− 0.25 to − 0.07)	− 2.88 (− 3.27 to − 2.48)
Smoking	1.31 (0.98 to 1.79)	0.08 (0.04 to 0.16)	1.16 (0.83 to 1.49)	0.06 (0.03 to 0.1)	− 0.69 (− 0.88 to − 0.49)	− 0.5 (− 0.68 to − 0.33)	− 2.06 (− 2.73 to − 1.38)
Secondhand smoke	0.12 (0.03 to 0.23)	0.28 (0.09 to 0.5)	0.11 (0.03 to 0.21)	0.14 (0.04 to 0.24)	− 2.07 (− 2.33 to − 1.82)	− 0.34 (− 0.44 to − 0.25)	− 3.13 (− 3.5 to − 2.76)
Alcohol use	0.17 (0.03 to 0.32)	–	0.21 (0.07 to 0.38)	–	− 0.21 (− 0.4 to − 0.03)	0.85 (0.59 to 1.11)	–
Low physical activity	0.05 (0.01 to 0.28)	0.05 (0 to 0.23)	0.06 (0.01 to 0.31)	0.03 (0 to 0.14)	− 0.84 (− 1.14 to − 0.54)	0.34 (0.11 to 0.58)	− 2.41 (− 2.84 to − 1.97)
Environmental/occupational risks	1.82 (1.37 to 2.47)	1.3 (0.99 to 1.68)	1.46 (1 to 1.9)	0.59 (0.43 to 0.78)	− 1.58 (− 1.75 to − 1.42)	− 0.64 (− 0.75 to − 0.53)	− 3.26 (− 3.63 to − 2.88)
Ambient particulate matter pollution	0.54 (0.24 to 0.96)	0.3 (0.13 to 0.53)	1.06 (0.71 to 1.41)	0.4 (0.28 to 0.55)	1.93 (1.61 to 2.25)	2.59 (2.34 to 2.85)	0.6 (0.05 to 1.15)
Household air pollution from solid fuels	1.04 (0.64 to 1.54)	0.86 (0.59 to 1.19)	0.22 (0.09 to 0.4)	0.13 (0.06 to 0.21)	− 6.02 (− 6.36 to − 5.69)	− 5.27 (− 5.51 to − 5.03)	− 7.08 (− 7.59 to − 6.57)
Lead exposure	0.26 (0.14 to 0.42)	0.11 (0.04 to 0.19)	0.1 (0.03 to 0.19)	0.02 (0 to 0.06)	− 3.69 (− 4.01 to − 3.36)	− 3.06 (− 3.36 to − 2.76)	− 5.44 (− 5.97 to − 4.92)
High temperature	0 (0 to 0.01)	0 (0 to 0.01)	0 (0 to 0.01)	0 (0 to 0.01)	0.31 (− 0.37 to 0.99)	1.22 (0.56 to 1.88)	− 1.29 (− 2.1 to − 0.48)
Low temperature	0.3 (0.2 to 0.44)	0.22 (0.15 to 0.3)	0.26 (0.17 to 0.38)	0.11 (0.07 to 0.15)	− 1.39 (− 1.56 to − 1.23)	− 0.44 (− 0.57 to − 0.31)	− 3.08 (− 3.45 to − 2.71)

No., number; ASMR, age-standardized mortality rate; ASDR, age-standardized disability-adjusted life years rate; EAPC, estimated annual percentage change; CI, confidence interval.

The ASDR for ischemic stroke in young adults was 187.7 per 100,000 population in 1990 and 155.38 per 100,000 population in 2019, and EAPC showed a decreasing trend from 1990 to 2019 [EAPC = − 0.86 (− 1 to − 0.72)] (Table S1). The EAPC of ASDR for metabolic risks, behavioral risks, and environmental/occupational risks was − 0.47 (− 0.6 to − 0.34), − 0.82 (− 0.94 to − 0.69), and − 1.27 (− 1.41 to − 1.14), respectively (Table 2 and Fig. 1B).

**Table 2:** Trends in ASDR for ischemic stroke in young adults in China from 1990 to 2019 for all risk factors.

	1990		2019		EAPC		
	ASDR for male per 10^5^ (95% CI)	ASDR for female per 10^5^ (95% CI)	ASDR for male per 10^5^ (95% CI)	ASDR for female per 10^5^ (95% CI)	Both No. (95% CI)	Male No. (95% CI)	Female No. (95% CI)
All risk factors	173.41 (136.65 to 225.58)	169.48 (132.65 to 213.33)	167.04 (127.43 to 206.54)	120.86 (88.98 to 156.58)	− 0.8 (− 0.94 to − 0.67)	− 0.12 (− 0.2 to − 0.04)	− 1.64 (− 1.88 to − 1.39)
Metabolic risks	133.72 (95.44 to 183.88)	133.4 (94.6 to 181.04)	143.25 (104.16 to 183.79)	103.06 (69.91 to 140.73)	− 0.47 (− 0.6 to − 0.34)	0.26 (0.19 to 0.33)	− 1.35 (− 1.6 to − 1.1)
High fasting plasma glucose	17.39 (4.62 to 38.81)	15.31 (3.77 to 36.09)	22.26 (5.9 to 47.79)	14.98 (3.93 to 33.78)	0.55 (0.48 to 0.62)	1.21 (1.06 to 1.36)	− 0.3 (− 0.47 to − 0.13)
High systolic blood pressure	73.12 (29.28 to 129.35)	64.45 (17.27 to 129.35)	93.92 (44.45 to 143.13)	58.18 (18.09 to 104.86)	0.19 (0.05 to 0.34)	0.92 (0.82 to 1.02)	− 0.8 (− 1.06 to − 0.53)
High LDL cholesterol	92.23 (59.55 to 128.48)	95.8 (62.35 to 133.16)	92.85 (59.81 to 128.42)	70.01 (44.57 to 98.73)	− 0.7 (− 0.84 to − 0.56)	0.04 (− 0.04 to 0.11)	− 1.55 (− 1.8 to − 1.31)
High body-mass index	26.35 (5.79 to 62.6)	28.71 (6.36 to 66.78)	58.4 (25.18 to 100.06)	39.22 (14.63 to 73.67)	1.89 (1.77 to 2.02)	2.92 (2.83 to 3)	0.67 (0.42 to 0.92)
Kidney dysfunction	14.75 (10.2 to 20.85)	19.25 (13.28 to 26.76)	14.43 (9.62 to 20.12)	13.62 (9.2 to 19.55)	− 0.62 (− 0.71 to − 0.54)	0.15 (0.04 to 0.25)	− 1.36 (− 1.52 to − 1.19)
Behavioral risks	143.78 (111.5 to 186.29)	116.78 (85.96 to 153.04)	136.36 (103.28 to 170.26)	81.35 (57.44 to 109.02)	− 0.82 (− 0.94 to − 0.69)	− 0.2 (− 0.27 to − 0.12)	− 1.71 (− 1.95 to − 1.47)
Diet low in fruits	26.3 (6.95 to 48.64)	26.98 (6.94 to 50.18)	18.87 (4.08 to 37.83)	14.05 (2.94 to 28.38)	− 1.95 (− 2.11 to − 1.78)	− 1.25 (− 1.35 to − 1.15)	− 2.77 (− 3.04 to − 2.5)
Diet low in vegetables	8.38 (0.68 to 16.81)	8.16 (0.61 to 16.32)	0.54 (0.29 to 1.41)	0.36 (0.2 to 0.87)	− 10.89 (− 11.55 to − 10.23)	− 10.14 (− 10.73 to − 9.55)	− 11.86 (− 12.62 to -11.1)
Diet high in red meat	28.13 (7.95 to 47.9)	28.88 (8.17 to 48.39)	44.18 (23.73 to 62.52)	33.23 (17.21 to 48.76)	1.11 (1.02 to 1.2)	1.83 (1.73 to 1.94)	0.26 (0.08 to 0.45)
Diet low in fiber	21.75 (4.72 to 41.96)	23.55 (5.25 to 42.98)	11.76 (1.55 to 29.03)	33.23 (17.21 to 48.76)	− 2.66 (− 2.83 to − 2.49)	− 2.06 (− 2.17 to − 1.95)	− 3.3 (− 3.57 to − 3.03)
Diet high in sodium	50.73 (20.63 to 89.17)	39.12 (14.29 to 71.97)	46.36 (18 to 81.66)	25.8 (8.22 to 48.94)	− 0.96 (− 1.09 to − 0.84)	− 0.31 (− 0.39 to − 0.23)	− 1.95 (− 2.2 to − 1.7)
Diet low in whole grains	21.49 (4.88 to 34.55)	21.49 (4.99 to 34.42)	20.95 (4.65 to 33.63)	15.5 (3.56 to 25.09)	− 0.78 (− 0.92 to − 0.64)	− 0.09 (− 0.16 to − 0.01)	− 1.6 (− 1.85 to − 1.35)
Smoking	77.22 (59.73 to 101.96)	6.09 (2.79 to 11.87)	71.9 (54.26 to 90.31)	6.23 (3.23 to 10.74)	− 0.53 (− 0.7 to − 0.35)	− 0.42 (− 0.57 to − 0.26)	− 0.7 (− 1.2 to − 0.2)
Secondhand smoke	6.59 (1.87 to 13.16)	20 (6.53 to 35.41)	6.13 (1.73 to 12.3)	12.5 (4.02 to 22.15)	− 1.58 (− 1.79 to − 1.37)	− 0.28 (− 0.37 to − 0.19)	− 2.19 (− 2.47 to − 1.91)
Alcohol use	10.45 (1.54 to 19.97)	–	14 (4.45 to 24.25)	–	1.25 (0.94 to 1.56)	1.01 (0.72 to 1.29)	–
Low physical activity	3.27 (0.38 to 18.79)	4.43 (0.42 to 21.64)	3.92 (0.37 to 22.07)	4.22 (0.3 to 17.78)	− 0.3 (− 0.65 to 0.05)	0.43 (0.17 to 0.7)	− 0.96 (− 1.4 to − 0.52)
Environmental/occupational risks	112.35 (87.04 to 146.15)	109.12 (83.4 to 137.26)	92.82 (68.13 to 118.01)	66.72 (49.15 to 87.61)	− 1.27 (− 1.41 to − 1.14)	− 0.59 (− 0.67 to − 0.51)	− 2.11 (− 2.36 to − 1.86)
Ambient particulate matter pollution	34.23 (15.25 to 59.84)	25.98 (11.73 to 46.96)	69.73 (49.83 to 90.63)	48.53 (33.94 to 66.16)	2.34 (2.09 to 2.58)	2.67 (2.47 to 2.88)	1.9 (1.56 to 2.25)
Household air pollution from solid fuels	65.41 (40.98 to 93.28)	74.96 (51.45 to 100.87)	13.89 (5.95 to 25.39)	14.45 (7.31 to 24.43)	− 5.64 (− 5.93 to − 5.35)	− 5.25 (− 5.47 to − 5.02)	− 6.03 (− 6.39 to − 5.66)
Lead exposure	15.78 (8.3 to 25.54)	8.87 (3.46 to 16.29)	5.91 (1.87 to 11.92)	2.4 (0.33 to 6.13)	− 3.72 (− 4 to − 3.44)	− 3.18 (− 3.45 to − 2.91)	− 4.78 (− 5.14 to − 4.41)
High temperature	0.1 (− 0.14 to 0.6)	0.07 (− 0.1 to 0.36)	0.12 (− 0.11 to 0.59)	0.05 (− 0.05 to 0.24)	0.35 (− 0.34 to 1.04)	1.28 (0.62 to 1.94)	− 1.25 (− 2.07 to − 0.43)
Low temperature	13.8 (9.43 to 20.37)	10.16 (6.96 to 14.26)	12.37 (7.94 to 17.67)	5.06 (3.41 to 7.24)	− 1.4 (− 1.58 to − 1.23)	− 0.43 (− 0.56 to − 0.31)	− 3.1 (− 3.48 to − 2.72)

No., number; ASDR, age-standardized disability-adjusted life years rate; EAPC, estimated annual percentage change; CI, confidence interval.

Females have the lower ASMR and ASDR and a faster decline (or lower increasing) trend in general. The EAPC for all risk factors, behavioral risks, and environment/occupational risks showed a decreasing trend for ASMR and ASDR for both males and females, but for metabolic risks, a decreasing trend for ASMR and ASDR for females [EAPC and 95% CI − 2.61 (− 2.99 to − 2.22) and − 1.35 (− 1.6 to − 1.1), respectively] and an increasing trend for males [EAPC and 95% CI 0.16 (0.06 to 0.25) and 0.26 (0.19 to 0.33), respectively] (Tables 1 and 2 and Fig. 1).

### Burden of detailed metabolic risk varied by sex

The results on detailed metabolic risk factors are interesting. ASMR for high fasting plasma glucose and high body-mass index showed an increasing trend [EAPC and 95% CI 0.09 (0.01 to 0.18) and 1.62 (1.45 to 1.79), respectively]. ASMR for high LDL cholesterol and kidney dysfunction displayed a declined trend [the EAPC and 95% CI − 1.05 (− 1.21 to − 0.89) and − 1.01 (− 1.13 to − 0.89), respectively]. The 95% CI of the EAPC for high systolic blood pressure contains 0.

For sex differences, there was a decreasing trend in ASMR for females for all detailed metabolic risk factors (the EAPC and 95% CI were under 0) (Table 1 and Fig. 1A), but an increasing trend for males for high fasting plasma glucose, high systolic blood pressure, and high body-mass index, especially the high body-mass index, with the EAPC and 95% CI of 2.84 (2.73 to 2.95). ASMR changes were not statistically significant for high LDL cholesterol and kidney dysfunction. ASDR for high body-mass index was increased both in males and females (the EAPC and 95% CI were greater than 0), which is the only one increased in metabolic risks both in males and females.

### Burden of detailed behavioral risk and the sex difference

Except for the risk of diet high in red meat, the burden of ischemic stroke attributable to the other dietary risk factors showed a declined trend for both males and females in China, and the decline trend was steeper for females than for males. High red meat intake was the only dietary risk factor associated with an increased burden of ischemic stroke. ASMR shows a decreasing trend for females but an increasing trend for males, and ASDR shows an increasing trend for both males and females.

From 1990 to 2019, the burden of ischemic stroke attributable to smoking and secondhand smoke decreased for both males and females in China. There is a lack of data on ischemic stroke attributable to alcohol use in females. For males, both ASMR and ASDR attributable to alcohol use have increased over the past three decades (Fig. 1, Tables 1 and 2). The burden of ischemic stroke attributable to low physical activity has increased for males over the past three decades, but decreased for female (Fig. 1 and Table 1).

### Burden of detailed environmental/occupational risks and the sex difference

The burden of ischemic stroke attributable to household air pollution from solid fuels, lead exposure, and low temperature decreased for both males and females. Both ASMR and ASDR attributable to ambient particulate matter pollution have increased significantly over the past three decades for both males and females. The burden attributable to high temperature increased in males but decreased in females (Fig. 1 and Tables 1 and 2).

### The main risk factors for the mortality of ischemic stroke varied between males and females

**Figure 2:**
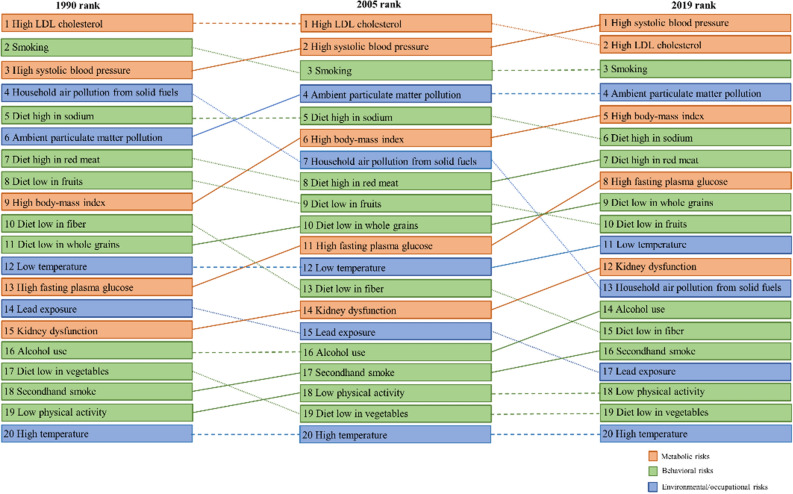
Rank of detailed risk factors for ischemic stroke mortality in males in 1990, 2005, and 2019.

**Figure 3:**
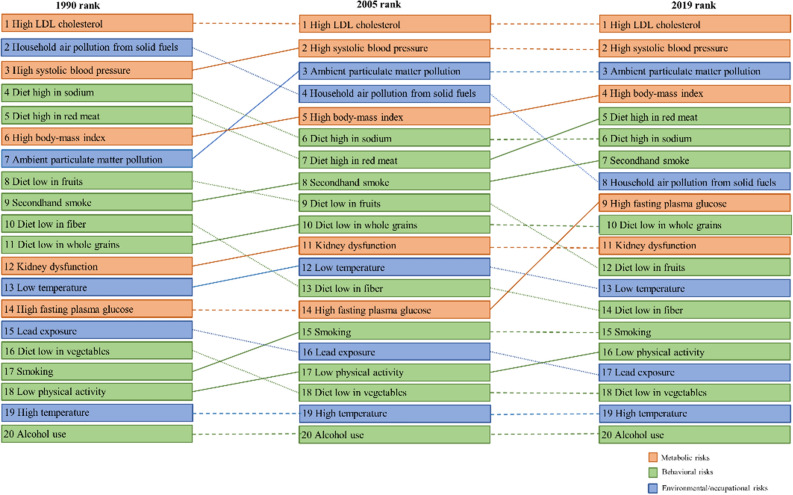
Rank of detailed risk factors for ischemic stroke mortality in females in 1990, 2005, and 2019.

Figures 2 and 3 showed the ASMR attributable to 20 detailed risk factors in descending order in 1990, 2005, and 2019 for male and female adults in China, respectively. Figures S1 and S2 showed the ASMR attributable to 20 detailed risk factors in descending order in 1990 and their trends from 1990 to 2019 in heatmap format for males and females Chinese adults, respectively. The primary risk factor for stroke mortality in males changed from high LDL cholesterol in 1990 to high systolic blood pressure in 2019. The ASMR attributable to high systolic blood pressure had an obvious growth from 1990 to 2019 (Figure S1). Smoking is one of the major risk factors for stroke mortality in young males in China. High LDL cholesterol has always been the major risk factor for ischemic stroke mortality in young females, but the trend of ASMR decline from 1990 to 2019 (Figure S2). High body-mass index and ambient particulate matter pollution became major risk factors in 2019 compared to 1990, both in males and females (Figs. 2 and 3). On the contrary, ischemic stroke mortality due to household air pollution from solid fuels decreased significantly both in males and females.

### Projection of the disease burden for ischemic stroke attributable to several specific risks

**Figure 4:**
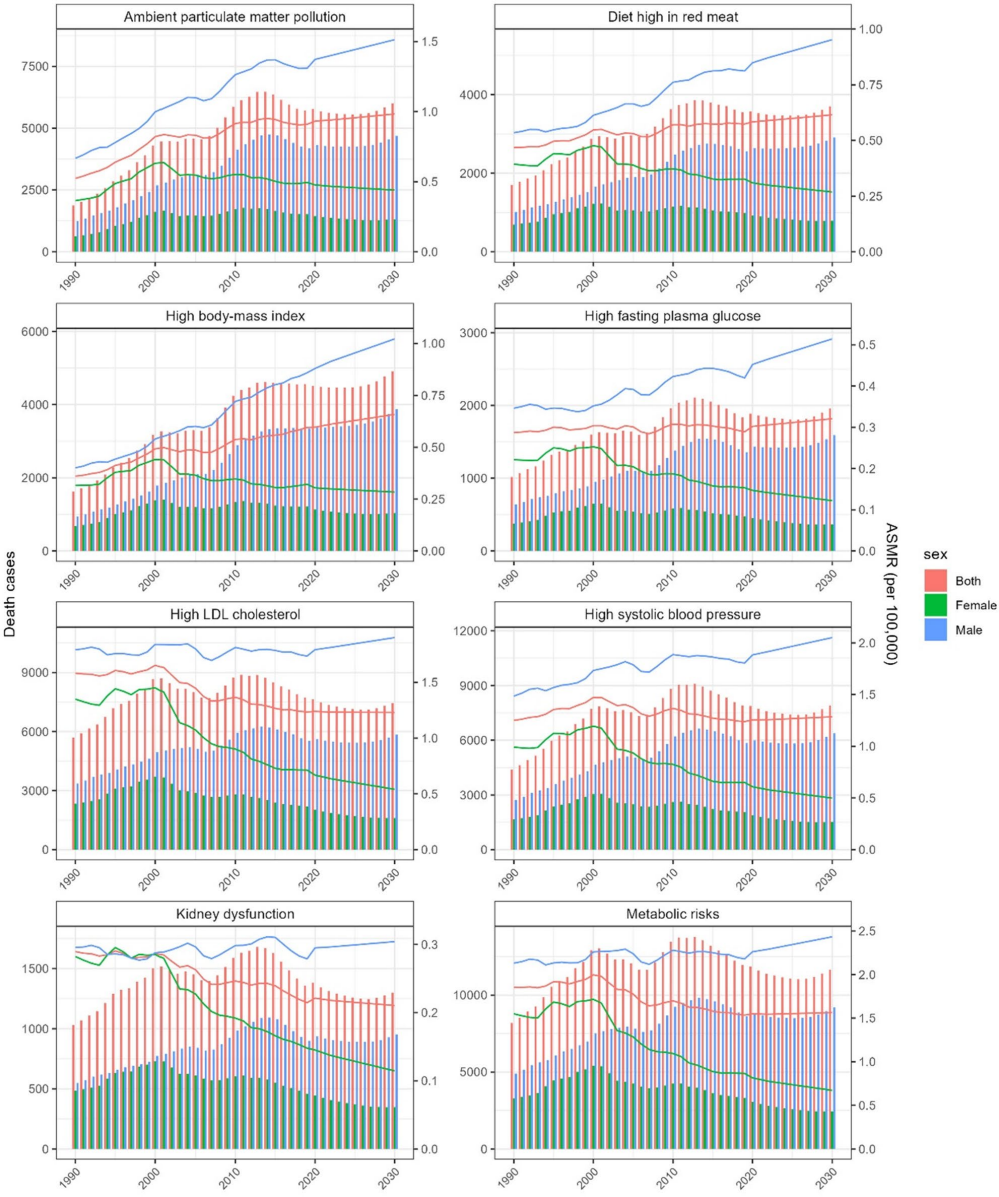
Projections of ASMR on ischemic stroke due to several risk factors in Chinese young adults aged 20–49 years from 2019 to 2030.

Based on the results presented above, we performed a projection of the factors that are likely to lead an increase in the burden of ischemic stroke over the next decade. Figure 4 showed the predicted disease burden of ischemic stroke due to metabolic and specific metabolic risks, high red meat intake, and ambient particulate matter pollution. Our results showed that the mortality rate for ischemic stroke attributable to these factors in young adults in China over the next 10 years is significantly different between males and females. ASMR for ischemic stroke attributable to ambient particulate matter pollution, which is the only one for females showed a slight increase from 1990 to 2030. But all seven specific risk factors, including high systolic blood pressure, high LDL cholesterol, high body-mass index, high fasting plasma glucose, kidney dysfunction, high red meat intake, and ambient particulate matter pollution, showed increasing trend over the next 10 years for males, especially high body-mass index, high red meat intake and ambient particulate matter pollution.

## Discussion

In this study, we systematically analyzed the deaths and DALYs, as well as relative risk factors for ischemic stroke in young Chinese adults from 1990 to 2019. We analyzed the trend of disease burden due to relevant risk factors over time for different sexualities. The disease burden associated with ischemic stroke in young adults in China is higher than the global but has been on a downward trend between 1990 and 2019. The trends of ASMR and ASDR were heterogeneous across sexes and different risk factors. Prevention and strategies to control the disease burden of ischemic stroke attributable to specific risk factors for different sexualities in Chinese adults are needed.

We find that the burden of ischemic stroke was heavier for males than for females, and has a more slowly decline in Chinese adults from 1990 to 2019. Relevant study about the global burden of ischemic stroke in young adults found that the ASDR and ASMR were higher in males than in females, which is consistent with our study^6^. Previous studies have shown that stroke affects females more than males^18^, contradicting our findings. We considered that this might be attributed to the protective effect of estrogen, which plays a significant role in adulthood and gradually decreases during menopause^19^. The level of economic development was also one of the factors affecting the prognosis of ischemic stroke. Ischemic stroke rates were lower in regions with high sociodemographic index than in regions with low sociodemographic index^6^. With China’s rapid economic development and changing perceptions, the educational inequality of Chinese women has improved, which may be the probable explanation for the steeper decline of ischemic stroke in Chinese female adults^20,21^.

Our study found that the disease burden of ischemic stroke due to metabolic risks declined the most slowly among the three risk categories. The ASMR of ischemic stroke attributable to high body-mass index for males was the fastest-growing of all detailed risks. The association between body-mass index and stroke is widely accepted^22,23^. Since the 1990s, the prevalence of overweight and obesity in China has been steadily increasing, and China now has the highest number of overweight and obese people^24,25^. Strokes caused by overweight and obesity have been a big burden in China. It is necessary to improve China’s policy system for obesity prevention and control. In addition to high body-mass index, the burden of ischemic stroke attributable to high fasting plasma glucose and systolic blood pressure has also been on the rise for males over the past three decades. The ASMR attributable to high systolic blood pressure for males rose from third in 1990 to first in 2019 (Figure S1). Hypertension and high plasma glucose were considered as modifiable risk factors for stroke onset and prognosis in general^26–28^. Previous study revealed that 23.2% (≈ 244.5 million) of the Chinese adults population ≥ 18 years of age had hypertension, and another 41.3% (≈ 435.3 million) had pre-hypertension according to the Chinese guideline^29^. The estimated prevalence of diabetes in China increased from 10.9% in 2013 to 12.4% in 2018, and the prevalence of prediabetes in China was 35.7% in 2013 and 38.1% in 2018^30^. The burden of ischemic stroke in young Chinese males due to metabolic factors has increased over the past three decades. China should develop more policies aimed at improving the metabolic status of young people to reduce the burden of stroke.

With the rapid development of economy, food shortages have improved significantly in China over the past three decades. Our study found that the disease burden due to behavioral risks generally decreased, especially for dietary changes in vegetables, fruits, sodium, etc. High red meat intake was the only dietary factor that has increased mortality, possibly due to urbanization and economic growth^31^. Another study found that stroke mortality attributable to high red meat intake showed a decreasing trend for female individuals in China, while it was stable for male individuals in China^32^. The burden of alcohol-induced ischemic stroke in males is also on the rise in China, probably due to the sharp increase in Chinese per-capita consumption^33^. The Chinese government should develop a national action plan with a public health perspective to prevent and reduce the damage caused by alcohol abuse in the workforce.

Disease burden of ischemic stroke due to environmental/occupational risks showed potential relationships with economic and industrial development. The burden of ischemic stroke among Chinese adults attributable to household air pollution from solid fuels has decreased significantly over the past three decades, while the burden of ischemic stroke attributable to ambient particulate matter pollution has risen sharply. With the progress of urbanization and industrial development, the living environment of Chinese residents has improved significantly, but at the same time, environmental pollution has come with it, which has led to the changes described above. Concerns are growing about the impact of ambient particulate matter pollution on stroke^34–36^, and all evidence suggests that reductions in air pollutant concentrations represent a significant population-level opportunity to reduce stroke risk. To reduce the disease and social burden caused by environmental particulate pollution, we suggest that China should more urgently formulate and enforce regulations on environmental pollution management.

Our projections suggest that the burden of ischemic stroke in young adults in China due to the seven specific risk factors will be significantly higher in males than females over the next 10 years. In addition to specific metabolic risk factors, ASMR for ischemic stroke due to red meat intake and ambient particulate matter pollution is significantly higher in males than females. One possible cause could be hormones. Estrogen plays a key role in regulating body weight, energy expenditure, and metabolic homeostasis^37^, and it is a protective factor against disease in females, especially before menopause^38^. Women had a healthier lifestyle such as lower sodium intake (4.5 versus 4.9g/d sodium) than men, which may be another reason for the sex differences^39^. In addition, we suggest that it may also be related to the nature of male work and greater social pressure.

This study illuminated the temporal trends and sex differences in the burden of ischemic stroke disease in young adults in China due to different risk factors over the past three decades, and predicted the disease burden due to several risk factors over the next 10 years with satisfactory quality. There are also several limitations. First, our data are not derived from primary data, which may increase bias. However, the GBD 2019 study used the Cause of Death Ensemble modeling framework to estimate deaths due to stroke subtypes and minimize the bias. Second, this study used a global perspective to analyze temporal trends in the risk factors for ischemic stroke in young Chinese adults, which may not be useful for individual guidance of clinical patients. Third, because ischemic stroke occurs primarily in older age groups, the attributable mortality rate is lower in young adults, but our focus is not only on the disease burden, but also on the temporal trends and workforce loss. Forth, we only discussed the common risk factors for ischemic stroke based on GBD database, but failed to study some risk factors based on youth, such as aortic dissection, autoimmune diseases, etc., which will be our direction of future research.

## Conclusion

Our results provide strong evidence that the disease burden of ischemic stroke in young adults in China has been decreasing over the past three decades, but with varying patterns of change by related risk factors and sex. The disease burden of ischemic stroke attributable to high plasma glucose, high body-mass index, high red meat intake, and ambient particulate matter pollution is increased in the past 3 decades. And the high blood pressure, high LDL, and high sodium intake was decreased. The burden of ischemic stroke was greater in males than in females, and the contribution of metabolic factors was increasingly significant. Metabolic risk is the major cause of ischemic stroke in young males in China, with an increasing trend from 1990 to 2019. Metabolic risk factors showed an increasing trend over the next 10 years for males, especially high body-mass index, also as high red meat intake and ambient particulate matter pollution. It is recommended that measures should be taken to prevent ischemic stroke in young Chinese males, especially those due to metabolic risk factors, in order to reduce workforce loss and socio-economic burden.

### Supplementary Information

Supplementary Information.

## Data Availability

The datasets presented in this study can be found in online (http:// ghdx.healthdata.org/gbd-results-tool and https://ghdx.healthdata.org/record/ihme-data/global-population-forecasts-2017-2100).
