# Supplementary material for: Analysis and projections of disease burden for different risk factors and sexes of ischemic stroke in young adults in China
Source: Sci Rep. 2024 Jun 10;14:13339. doi: 10.1038/s41598-024-63920-0 (PMC11164860; doi:10.1038/s41598-024-63920-0)
Supplement: Supplementary file 1 — Supplementary Information. [file 41598_2024_63920_MOESM1_ESM.pdf]

# Analysis and projections of disease burden for different risk factors and sexes of ischemic stroke in young adults in China

Ying Gao[1], Kangding Liu [1], Shaokuan Fang [1*]

[1] Department of Neurology, Neuroscience Centre, the First Hospital of Jilin University, Changchun, China

*Corresponding author at: No. 1 Xinmin Street, Chaoyang District, Changchun, 130021,

Jilin Province, People's Republic of China.

E-mail address: fangsk@jlu.edu.cn

**Definition of risk factor**

Ambient PM2.5 pollution was defined as an annual average daily exposure to outdoor air concentrations of PM2.5 more than 8.8 μg/m3.

Household air pollution was defined as any exposure to indoor concentration of PM2.5.

Lead exposure was defined as blood lead concentration of more than 20 μg/L.

Diet high in sodium was defined as consumption of sodium more than 5 g/day.

Diet low in fruits was defined as consumption of less than 200 g/day.

Diet low in vegetables was defined as consumption of less than 350 g/day.

Diet low in whole grains was defined as consumption of less than 100 g/day.

Alcohol use was defined as any amount.

Low physical activity was defined as average weekly work, home, transport-related, and recreational physical activity of less than 8000 metabolic equivalent of task-min.

Smoking was defined as any previous or current tobacco smoking.

Second-hand smoking was defined as daily indoor exposure to second-hand smoking.

High body-mass index (BMI) was defined as body-mass index greater than 23.0 kg/m$^2$.

High fasting plasma glucose was defined as serum fasting plasma glucose greater than 5.4 mmol/L.

High systolic blood pressure (SBP) was defined as systolic blood pressure greater than 110-115 mm Hg.

High (low density lipoprotein) LDL cholesterol was estimated in units of mmol/L (we used a TMREL with a uniform distribution between 0·7 and 1·3mmol/L).

Low glomerular filtration rate was defined as glomerular filtration rate less than 60 mL/min per 1.73m$^2$ (excluding end-stage renal disease).

The exposure of non-optimal temperature is defined as the daily exposure to ambient temperature that is either warmer or colder than the temperature associated with the minimum mortality risk. Specifically, we define the theoretical minimum risk exposure level (TMREL) for temperature as the temperature that is associated with the lowest overall mortality attributable to the risk, in a given location and year. Given varying exposure-response curves for different mean annual temperature zones, as well as spatially and temporally varying cause compositions, we estimate TMRELs by year and location and are not using a globally uniform TMREL. High temperature (heat) exposure is defined as exposure to temperatures warmer than this TMREL and low temperature (cold) is defined as temperatures colder than this TMREL.

**ICD codes for stroke categories**

There were three main data sources for stroke-caused death that were included in GBD 2019 and reported with two versions of ICD-9 and ICD-10. The corresponding codes in International

Classification of Disease 10th revision (ICD-10) for each stroke category were shown in the table below.

**ICD codes for stroke categories**

| Stroke category | ICD-9 Codes | ICD-10 Codes |
|---|---|---|
| **Fatal analysis** | | |
| Ischemic stroke | 433-435.9, 437.0-437.1, 437.5 437.8 | G45-G46.8, I63-I63.9, I65-I66.9, I67.2-I67.3, I67.5-I67.6, I69.3 |
| Hemorrhagic stroke | 430-432.9, 437.2 | I60-I62.9, I67.0-I67.1, I68.1-I68.2, I69.0-I69.2 |
| **Non-fatal analysis** | | |
| Ischemic stroke | 433-434.9, 437.0-437.1, 437.5 437.8 | I63-I63.9, I65-I66.9, I67.2-I67.3, I67.5-I67.6, I69.3 |
| Hemorrhagic stroke | 430-432.9, 437.2 | I60-I62.9, I67.0-I67.1, I68.1-I68.2, I69.0-I69.2 |

Figure S1: Ischemic stroke ASMR attributable to 20 detailed risk factors in descending order for young adults aged 20-49 years in 1990 and their trends from 1990 to 2019 for Chinese males.

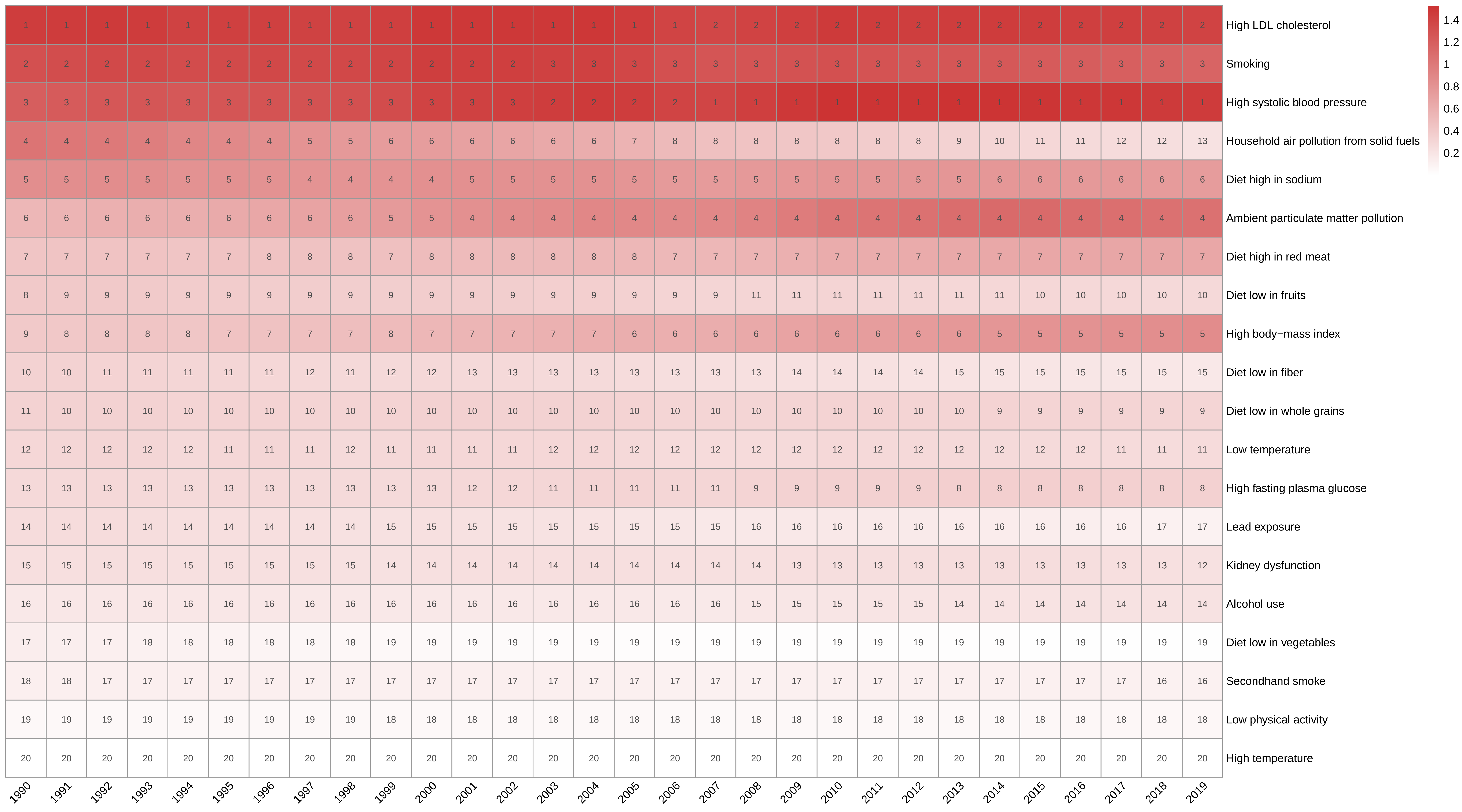

High LDL cholesterol 1 1 1 1 1 1 1 1 1 1 1 1 1 1 1 1 1 2 2 2 2 2 2 2 2 2 2 2 2 2
Smoking 2 2 2 2 2 2 2 2 2 2 2 2 2 3 3 3 3 3 3 3 3 3 3 3 3 3 3 3 3 3
High systolic blood pressure 3 3 3 3 3 3 3 3 3 3 3 3 3 2 2 2 2 1 1 1 1 1 1 1 1 1 1 1 1 1
Household air pollution from solid fuels 4 4 4 4 4 4 4 5 5 6 6 6 6 6 6 7 8 8 8 8 8 8 8 9 10 11 11 12 12 13
Diet high in sodium 5 5 5 5 5 5 5 4 4 4 4 5 5 5 5 5 5 5 5 5 5 5 5 5 6 6 6 6 6 6
Ambient particulate matter pollution 6 6 6 6 6 6 6 6 6 5 5 4 4 4 4 4 4 4 4 4 4 4 4 4 4 4 4 4 4 4
Diet high in red meat 7 7 7 7 7 7 8 8 8 7 8 8 8 8 8 8 7 7 7 7 7 7 7 7 7 7 7 7 7 7
Diet low in fruits 8 9 9 9 9 9 9 9 9 9 9 9 9 9 9 9 9 9 11 11 11 11 11 11 11 10 10 10 10 10
High body−mass index 9 8 8 8 8 7 7 7 7 8 7 7 7 7 7 6 6 6 6 6 6 6 6 6 5 5 5 5 5 5
Diet low in fiber 10 10 11 11 11 11 11 12 11 12 12 13 13 13 13 13 13 13 13 14 14 14 14 15 15 15 15 15 15 15
Diet low in whole grains 11 10 10 10 10 10 10 10 10 10 10 10 10 10 10 10 10 10 10 10 10 10 10 10 9 9 9 9 9 9
Low temperature 12 12 12 12 12 11 11 11 12 11 11 11 11 12 12 12 12 12 12 12 12 12 12 12 12 12 12 11 11 11
High fasting plasma glucose 13 13 13 13 13 13 13 13 13 13 13 12 12 11 11 11 11 11 9 9 9 9 9 8 8 8 8 8 8 8
Lead exposure 14 14 14 14 14 14 14 14 14 15 15 15 15 15 15 15 15 15 16 16 16 16 16 16 16 16 16 16 17 17
Kidney dysfunction 15 15 15 15 15 15 15 15 15 14 14 14 14 14 14 14 14 14 14 13 13 13 13 13 13 13 13 13 13 12
Alcohol use 16 16 16 16 16 16 16 16 16 16 16 16 16 16 16 16 16 16 15 15 15 15 15 14 14 14 14 14 14 14
Diet low in vegetables 17 17 17 18 18 18 18 18 18 19 19 19 19 19 19 19 19 19 19 19 19 19 19 19 19 19 19 19 19 19
Secondhand smoke 18 18 17 17 17 17 17 17 17 17 17 17 17 17 17 17 17 17 17 17 17 17 17 17 17 17 17 17 16 16
Low physical activity 19 19 19 19 19 19 19 19 19 18 18 18 18 18 18 18 18 18 18 18 18 18 18 18 18 18 18 18 18 18
High temperature 20 20 20 20 20 20 20 20 20 20 20 20 20 20 20 20 20 20 20 20 20 20 20 20 20 20 20 20 20 20
1990 1991 1992 1993 1994 1995 1996 1997 1998 1999 2000 2001 2002 2003 2004 2005 2006 2007 2008 2009 2010 2011 2012 2013 2014 2015 2016 2017 2018 2019
1.4
1.2
1
0.8
0.6
0.4
0.2

Figure S2: Ischemic stroke ASMR attributable to 20 detailed risk factors in descending order for young adults aged 20-49 years in 1990 and their trends from 1990 to 2019 for Chinese females.

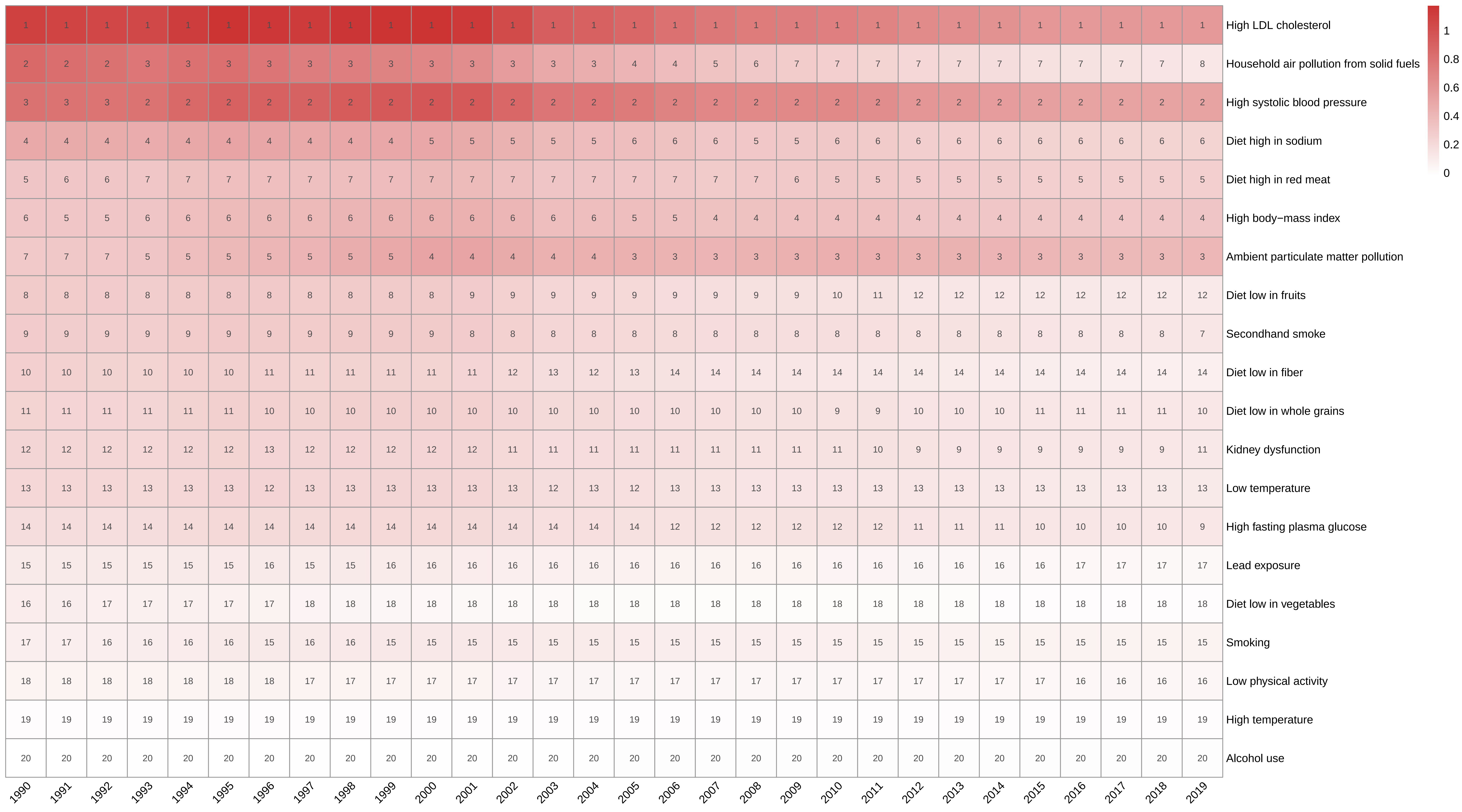
High LDL cholesterol
Household air pollution from solid fuels
High systolic blood pressure
Diet high in sodium
Diet high in red meat
High body−mass index
Ambient particulate matter pollution
Diet low in fruits
Secondhand smoke
Diet low in fiber
Diet low in whole grains
Kidney dysfunction
Low temperature
High fasting plasma glucose
Lead exposure
Diet low in vegetables
Smoking
Low physical activity
High temperature
Alcohol use
1990 1991 1992 1993 1994 1995 1996 1997 1998 1999 2000 2001 2002 2003 2004 2005 2006 2007 2008 2009 2010 2011 2012 2013 2014 2015 2016 2017 2018 2019
1
0.8
0.6
0.4
0.2
0

| | | 1990 | | 2019 | | EAPC | |
|---|---|---|---|---|---|---|---|
| | | ASMR per $10^5$ No. (95% CI) | ASDR per $10^5$ No. (95% CI) | ASMR per $10^5$ No. (95% CI) | ASDR per $10^5$ No. (95% CI) | ASMR No. (95% CI) | ASDR No. (95% CI) |
| China | Both | 2.52 (2.08 to 3.14) | 187.7 (153.68 to 230.27) | 1.87 (1.48 to 2.25) | 155.38 (123.95 to 189.54) | -1.23 (-1.39 to -1.07) | -0.86 (-1 to -0.72) |
| | Male | 2.89 (2.21 to 3.91) | 186.04 (147.25 to 240.36) | 2.63 (1.91 to 3.35) | 175.72 (135.28 to 216.51) | -0.27 (-0.35 to -0.18) | -0.19 (-0.27 to -0.11) |
| | Female | 2.1 (1.65 to 2.68) | 189.1 (148.62 to 235.35) | 1.07 (0.81 to 1.38) | 134.3 (100.39 to 172.54) | -2.93 (-3.31 to -2.55) | -1.65 (-1.9 to -1.4) |
| Global | Both | 1.92 (1.73 to 2.26) | 148.63 (126.33 to 175.66) | 1.53 (1.35 to 1.73) | 130.86 (109.27 to 155.04) | -0.96 (-1.09 to -0.84) | -0.56 (-0.62 to -0.49) |
| | Male | 2.19 (1.9 to 2.67) | 147.6 (124.93 to 175.41) | 1.86 (1.58 to 2.17) | 134.25 (112.36 to 158.13) | -0.66 (-0.79 to -0.54) | -0.39 (-0.45 to -0.32) |
| | Female | 1.63 (1.45 to 1.9) | 149.58 (123.7 to 179.67) | 1.19 (1.04 to 1.37) | 127.46 (102.94 to 155.08) | -1.39 (-1.54 to -1.24 | -0.73 (-0.82 to -0.63) |

Table S1: Trends of ASMR and ASDR for ischemic stroke in young adults in China and globally from 1990 to 2019.

No. number, ASMR age-standardized mortality rate, ASDR age-standardized disability-adjusted life years rate, EAPC estimated annual percentage change, CI confidence interval
